# Epithelioid Hemangioma Masquerading as a Clavicular Mass: A Diagnostic Conundrum

**DOI:** 10.7759/cureus.63449

**Published:** 2024-06-29

**Authors:** Pawani P Duddu, Inuganti Venkata Renuka, Santhi Imandi, Haritha Shah

**Affiliations:** 1 Pathology, NRI Medical College, Guntur, IND

**Keywords:** vascular tumour, hemangioma, clavicular swelling, bone tumors, epithelioid hemangioma

## Abstract

Epithelioid hemangioma (EH), a rare benign tumor, is documented in only a few cases in the medical literature. The complications of epithelioid hemangioma depend on the site of their presentation. If located in the eye, epithelioid hemangiomas may lead to amblyopia; in the larynx, they can cause respiratory issues; and if affecting bone, they can result in osteolytic lesions. Here, we present a case of a 40-year-old male who presented with swelling in the medial end of the right clavicle. Following clinical evaluation and a computed tomography scan, an excision biopsy was performed. The excised lesion measured 3 x 2.5 cm, and histopathological examination confirmed the finding as an epithelioid hemangioma.

## Introduction

Hemangiomas are rare benign vascular neoplasms most commonly found in the head and neck, primarily on the forehead, as well as in the oral cavity, bones, and lymph nodes [1]. They are characterized by blood vessels surrounded by eosinophilic, epithelioid endothelial cells, and various inflammatory cells [1]. Trauma to bones or blood vessels may trigger their development, suggesting a potential underlying pathology [2]. Although hemangiomas are rare and lack a strong predisposition, adults show a slight tendency to develop this condition [3]. The clinical presentation varies depending on the site and may include subcutaneous nodules, abdominal discomfort, bone fractures, or even respiratory difficulties [3]. In this case report, we outline the clinical progression, diagnosis, and treatment of a case of epithelioid hemangioma in a patient who presented with a swelling in the medial end of the right clavicle.

## Case presentation

A 40-year-old Indian male presented to the outpatient department with a painful swelling at the medial end of the right clavicle for one month. The patient had no record of any co-morbidities, such as diabetes or hypertension, and no history of any past surgeries. The patient was not on any medication prior to his visit to the hospital.

**Figure 1:**
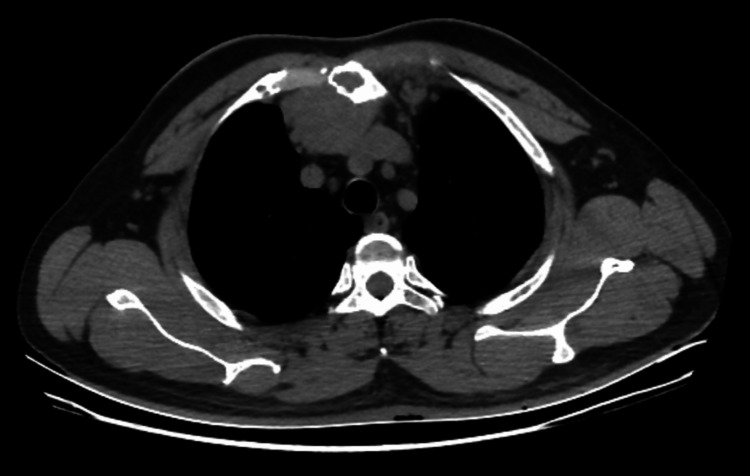
Contrast-enhanced computed tomography (CECT) of the chest A well-defined, heterogeneously enhanced soft tissue mass on the right side of the manubrium sterni.

During clinical examination, a tender, palpable mass was identified over the medial end of the right clavicle. Laboratory tests showed leukocytosis with peripheral eosinophilia, while all other laboratory results were within normal ranges. A contrast-enhanced computed tomography (CECT) of the chest showed a well-defined, heterogeneously enhanced soft tissue mass measuring 4.6 x 4.4 x 4 cm on the right side of the manubrium sternum (Figure 1). It extended cranio-caudally from the D1 to D4 vertebral body. A computed tomography (CT)-guided fine needle aspiration cytology (FNAC) was performed to confirm the findings observed in the CECT scan and support the diagnosis of epithelioid hemangioma.

**Figure 2:**
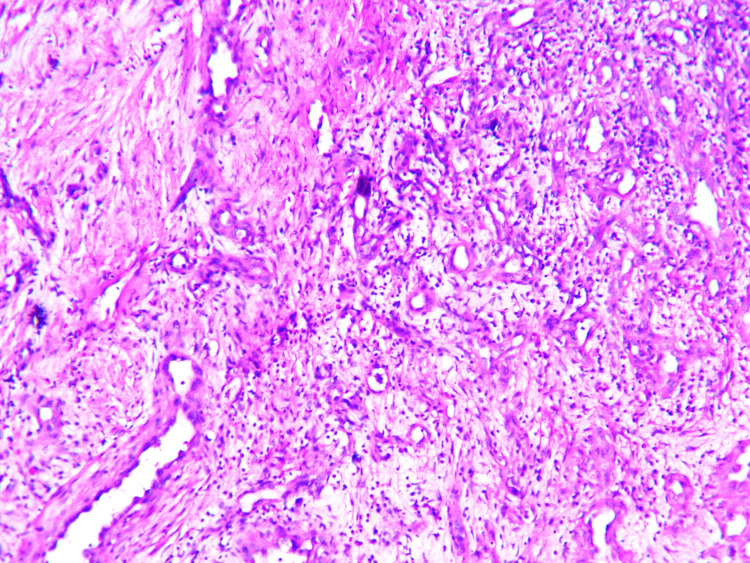
Microphotographs of epithelioid hemangioma Showing blood vessels and fibrous stroma (H&E scanner view).

**Figure 3:**
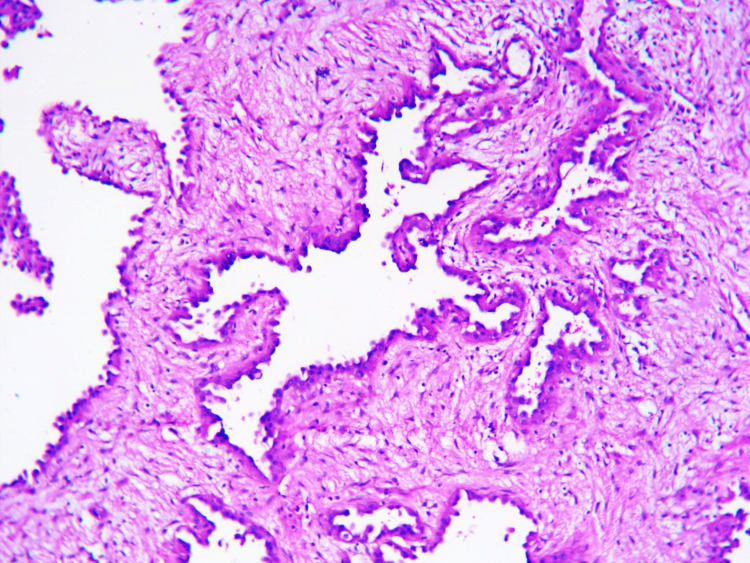
Microphotographs of epithelioid hemangioma Blood vessels lined by plump endothelial cells show scant fibrous tissue stroma (H&E x 100).

**Figure 4:**
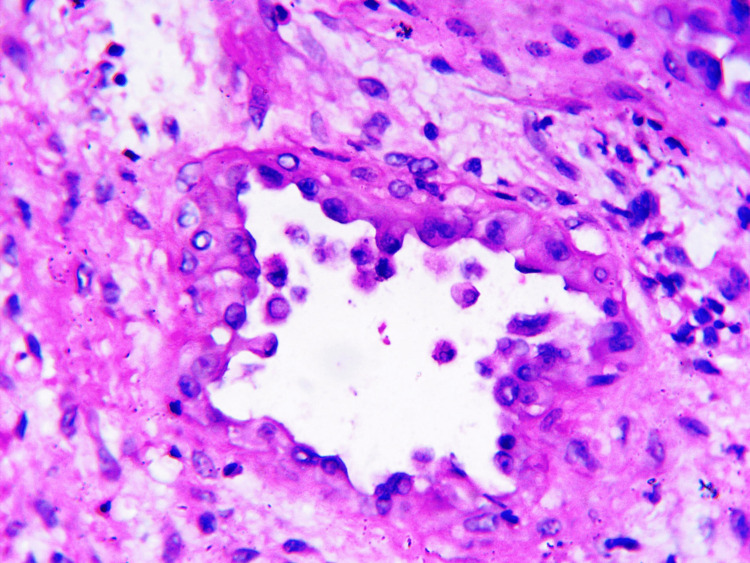
Microphotographs of epithelioid hemangioma Showing blood vessels lined by plump cells with vesicular nuclei and abundant eosinophilic cytoplasm (H&E x 400).

An excisional biopsy was performed on the lesion and sent for histopathological examination. On examination, a nodular, gray-brown lesion measuring 3 x 2.5 cm was identified, showing gray-white areas on the cut-section. Microscopically, minimal to no mitotic activity was observed, although a hypocellular and fibrous stroma was present (Figure 2). Additionally, scant connective tissue was observed in the stroma (Figure 3), and blood vessels were lined by plump cells with vesicular nuclei and abundant eosinophilic cytoplasm (Figure 4).

## Discussion

Epithelioid hemangioma was initially described as angiolymphoid hyperplasia with eosinophilia [1]. It can manifest in various sites, such as the eye, larynx, or bone [1]. The latter site was observed in our case. Based on recurrence and lymph node involvement, it can be classified as a more malignant or benign condition [4]. The tumor described in our case was benign in nature. Epithelioid hemangioma is characterized by blood vessels surrounded by plump, eosinophilic endothelial cells [1]. In our case, we observed blood vessels lined by plump cells with vesicular nuclei and abundant eosinophilic cytoplasm. These lesions may be localized to a single system or present at multiple sites simultaneously. In our case, there was a solitary lesion confined to the bone of the right clavicle.

Diagnosis involves a combination of clinical correlation, imaging, and biopsy, with biopsy considered the gold standard method [3]. In our case, clinical examination, coupled with contrast-enhanced computed tomography, revealed a well-defined, heterogeneously enhanced mass at the manubrium sternum (Table 1). These findings, combined with the biopsy report, correlated with the observations and led to the diagnosis of epithelioid hemangioma.

**Table 1:** Correlation of clinicopathological features with other studies M: male; F: female; MRI: magnetic resonance imaging; USG: ultrasound sonography; CECT: contrast-enhanced computed tomography

No.	Study	Age (years)/Sex	Size (cm)	Site	Clinical Presentation	Imaging Findings	Histopathology	Treatment
1	Ángeles et al. [5]	32/M	1.3x0.8	Humeral artery	Lump on the right mid-arm	MRI: nodule formation is seen close to humeral artery and vein; USG: hyperechoic nodular lesion having smooth edges in contact with brachial artery	The tumor shows high vascularity and is infiltrated by epithelioid cells	Bypass with basilic branch graft
2	Nangia et al. [6]	30/F	2x1	Lingual alveolar mucosa	Painful, progressive swelling in the lingual gum tissue	Radiographs: the alveolar bone remained undamaged and unaffected by the mass positioned above it	The connective tissue encircled by epithelioid cells alongside a dense presence of inflammatory cells around the vessels	Excision of lesion
3	Wiggins et al. [7]	16/M	15x5	Scapular region	Painless, growing mass over left scapula	USG: highly vascularised intramuscular mass; MRI: intramuscular mass, prominent blood vessels observed	Spindle-shaped cells and robust endothelial cells	Excision, tissue rearrangement
4	Present Case	40/M	3x2.5	Right clavicle	Painful swelling in the medial end of right clavicle	CECT chest: a well-defined heterogeneously enhanced mass at manubrium sternum	Lobular tumor composed of central epithelioid cells, and blood vessels lined by large oval cells with abundant eosinophilic cytoplasm and vesicular nuclei	Excision biopsy

The treatment approach for epithelioid hemangioma varies depending on the individual case. In some asymptomatic cases, simple monitoring of the lesion may be sufficient, as it can regress over time [4]. However, in symptomatic cases, procedures such as curettage, embolization, or excisional biopsy may be performed to mitigate potential complications. In our case, we opted for an excisional biopsy of the lesion. The patient did not report any postoperative complaints.

Two years later, the patient returned with similar complaints, and a histopathological examination confirmed a recurrence of epithelioid hemangioma without any features of malignancy. However, the patient was lost to follow up.

## Conclusions

We report a rare case of epithelioid hemangioma of bone in a patient who presented with a mass and associated tenderness at the medial end of the right clavicle. We opted for excisional biopsy as the treatment of choice, and the patient experienced no postoperative complications. However, the patient had a recurrence of epithelioid hemangioma with no features of malignancy two years later. The patient was subsequently lost to follow-up.
